# Study on the clinical efficacy and safety of baloxavir marboxil tablets in the treatment of influenza A

**DOI:** 10.3389/fmed.2024.1339368

**Published:** 2024-04-05

**Authors:** Chaochao Qiu, Fang Cheng, Xinchun Ye, Zhengxing Wu, Hongye Ning, Saiduo Liu, Lianpeng Wu, Yiyang Zhang, Jichan Shi, Xiangao Jiang

**Affiliations:** Department of Infection, Wenzhou Central Hospital, Dingli Clinical College of Wenzhou Medical University, Wenzhou, Zhejiang, China

**Keywords:** influenza, baloxavir marboxil tablets, oseltamivir, curative effect, adverse reactions

## Abstract

**Objective:**

To evaluate the clinical efficacy and safety of baloxavir marboxil tablets in the treatment of influenza A.

**Methods:**

According to a random sequence generated by computer software, 200 patients with confirmed influenza A were divided into a study group and a control group with 100 cases in each group. Group allocation was concealed using sealed envelopes. The study group was treated with oral administration of baloxavir marboxil tablets, 40 mg once. The control group was given oral oseltamivir capsules, 75 mg twice a day, for five consecutive days. The therapeutic effects, symptom disappearance time and adverse drug reactions of the two groups after 5 days of treatment were compared.

**Results:**

There was no significant difference in the total effective rate between the two groups (99% vs. 98%, *p* > 0.05). There was no significant difference in fever subsidence time (1.54 ± 0.66 d vs. 1.67 ± 0.71 d, *p* > 0.05), cough improvement time (2.26 ± 0.91 d vs. 2.30 ± 0.90 d, *p* > 0.05) and sore throat improvement time (2.06 ± 0.86 d vs. 2.09 ± 0.83 d, *p* > 0.05) between the two groups. There was no significant difference in the incidence of adverse drug reactions between the two groups (8% vs. 13%, *p* > 0.05).

**Conclusion:**

Baloxavir marboxil tablets can be effectively used in the treatment of patients with influenza A and have a similar efficacy and safety profile as oseltamivir capsules.

## 1 Introduction

Influenza is an acute respiratory infectious disease caused by the influenza virus (1). It has the characteristics of high incidence, rapid spread, a wide susceptible population and seasonality (2). Seasonal influenza epidemics can include 3–5 million severe cases worldwide each year, including 290,000–650,000 influenza-related respiratory disease-related deaths (3). If timely and effective treatment is not provided, serious complications can arise and threaten the safety of patients (4).

Anti-influenza virus treatment is an important means to control the virus. Oseltamivir is the most commonly used anti-influenza A virus drug at present. However, in 2017, the World Health Organization updated its “Standard List of Essential Drugs” to indicate that oseltamivir had not achieved its expected clinical efficacy when it was first added to the list in 2009. Therefore, it was moved from being a core drug to being an auxiliary drug, indicating that its use was limited to critically ill hospitalized patients with a confirmed or suspected influenza virus infection (5, 6). Baloxavir marboxil tablets (trade name Xofluza) are the world’s first FDA-approved RNA polymerase inhibitors for the treatment of simple acute influenza patients aged 12 years and older for no more than 48 h (7, 8). Compared with neuraminidase inhibitors, baloxavir has the advantages of fewer does, faster antiviral efficacy and fewer adverse reactions and can quickly relieve the dyspnea of adult patients with influenza A (9, 10). Studies have shown that baloxavir is equally effective for oseltamivir-resistant strains and avian influenza virus strains and can be used as an alternative therapy for oseltamivir resistance (11). However, for children under 12 years old, older people above the age of 65 years and patients with severe underlying diseases, its safety and effectiveness must be further confirmed by more clinical studies (12). In this study, we compared baloxavir with oseltamivir for treating patients with influenza A who were aged between 14 and 85 years old.

## 2 Materials and methods

### 2.1 Study design

This was a prospective, randomized and parallel-controlled trial conducted at Wenzhou Central Hospital from January to March 2022. The study protocol was approved by the Ethics Committee of Wenzhou Central Hospital (approval number: L2022-04-019). All participants gave written informed consent before being enrolled in the study and could withdraw at any time without penalty. The primary outcome was the time to symptom alleviation (TSA), which was defined as the time from the start of treatment until all symptoms (fever, cough, sore throat, nasal congestion, headache, myalgia and fatigue) were absent or very mild for at least 21.5 h. The secondary outcomes included the time to fever resolution (TFR), which was defined as the time from the start of treatment until the body temperature was below 37°C for at least 24 h; the time to viral clearance (TVC), which was defined as the time from the start of treatment until two consecutive negative results for influenza A virus nucleic acid test; and the incidence of adverse drug reactions (ADRs), which were recorded according to the World Health Organization’s definition and classification.

### 2.2 Study participants

A convenient sampling method was used to select 200 patients diagnosed in the fever clinic of Wenzhou Central Hospital as the study participants. The inclusion criteria were: (1) admission body temperature ≥ 37.3°C; varying degrees of headache, sore throat, nasal congestion, cough, myalgia, sweating, chills or fatigue and other symptoms; (2) within 48 h of onset; (3) novel coronavirus nucleic acid test was negative within 24 h; (4) aged 14–85 years. The exclusion criteria were: (1) combined with other serious organic diseases (e.g., heart disease, kidney disease, liver disease, gastrointestinal disease, mental disorders and the use of immunosuppressive agents); (2) pregnant or lactating women; (3) patients who were taking other antiviral drugs before enrolment; (4) patients who were intolerant to the test drug; (5) bacterial infection; (6) positive influenza B virus nucleic acid test; (7) cases diagnosed as severe or critical influenza.

### 2.3 Diagnostic standard

The diagnosis of influenza A was based on the diagnostic criteria of the National Health Commission’s influenza diagnosis and treatment plan (2018 revised edition) (1) and a positive throat swab result for an influenza A virus nucleic acid test. The test was performed using a real-time PCR kit (Shanghai BioGerm Medical Biotechnology Co., Ltd., batch number: BG20210001) with a sensitivity of 98% and a specificity of 99%. The cut-off value was 0.1 ng/mL. The diagnostic criteria for severe and critical cases were as follows. 1. Severe cases: (1) continuous high fever >3 days, accompanied by severe cough, purulent sputum, blood sputum or chest pain; (2) rapid breathing, dyspnea, cyanosis of the lips; (3) mental changes, e.g., slow response, lethargy, restlessness and convulsions; (4) severe vomiting, diarrhea and dehydration; (5) combined pneumonia; (6) original basic diseases were significantly aggravated. 2. Critical cases: (1) respiratory failure; (2) acute necrotizing encephalopathy; (3) septic shock; (4) multiple organ dysfunction; (5) other serious clinical situations requiring intensive care.

### 2.4 Randomization and blinding

According to a random sequence generated by computer software (SPSS, v.19.0), the patients were divided into a study group and a control group, with 100 cases in each group. The group allocation was concealed using sealed envelopes that contained the group assignment and were opened by a nurse who was not involved in the study after the patients consented to participate. The patients and the outcome assessors were blinded to the group allocation using identical-looking tablets for both groups.

Intervention: The patients in both groups were treated with multiple meals, nutritional support and rest. When the body temperature was >38.5°C, oral antipyretics (ibuprofen suspension, 10 mL) were given. The control group was given oral oseltamivir capsules (Tamiflu) (F. Hoffmann-La Roche Ltd., batch number: H20090377; h20090565) 75 mg twice a day, for five consecutive days. The study group was treated with oral administration of baloxavir tablets (Xofluza) (Shionogi Pharma Co. Ltd., batch number: HJ20210027) 40 mg once and followed up for another 4 days, the study group received placebo pills twice a day for the remaining 4 days to maintain the blinding of the study.

### 2.5 Assessment of treatment effect

The therapeutic effect was evaluated according to three categories, i.e., markedly effective, effective and ineffective. Markedly effective: the patient’s body temperature dropped to normal level after 48 h of medication, and symptoms such as cough and sore throat essentially or completely disappeared; effective: the patient’s body temperature returned to normal after 72 h of medication with cough, sore throat and other symptoms alleviated; ineffective: no significant change in body temperature after 72 h of medication and symptoms such as cough and sore throat were not improved or even aggravated. Total effective rate = (markedly effective cases + effective cases) /total cases × 100%.

### 2.6 Observed indexes

After 5 days of treatment, the therapeutic effects of the two groups were compared. The disappearance time of influenza symptoms (fever, cough, sore throat) was compared between the two groups. The adverse drug reactions of the two groups were also compared. The symptoms, adverse reactions, and other information were collected by nurses who interviewed the patients face-to-face at baseline and at day 5 after treatment initiation. In our study, we characterize “history of underlying diseases” as the presence of any enduring health conditions that could potentially impact the immune system or influence the individual’s response to an influenza infection or treatment. Such conditions encompass, but are not limited to, hypertension, diabetes, rheumatoid arthritis, and so forth.

### 2.7 Statistical method

The SPSS (v.19.0) software was used for data processing. Quantitative data were described by mean ± standard deviation, and a t-test was used for comparison between groups. Qualitative data were described by the number of cases or as a percentage, and a chi-square test or Fisher’s exact test was used for comparison between groups as appropriate; *p* < 0.05 was considered statistically significant.

## 3 Results

### 3.1 General information of enrolled patients

The time from onset to drug administration was 23.4 ± 6.7 h in the control group and 22.6 ± 7.2 h in the study group (*t* = 0.798, *p* = 0.426). The patient’s condition at onset was similar between the two groups, with no significant difference in body temperature (38.7 ± 0.5°C vs. 38.6 ± 0.6°C, *t* = 1.214, *p* = 0.226). In the control group, there were 48 men and 52 women, aged 14–80 years, with an average age of 44.1 ± 16.9 years. Among them, 8 patients had a history of underlying diseases, 6 had a history of hypertension, 1 had a history of diabetes, and 1 had Hashimoto’s thyroiditis. There were 51 men and 49 women in the study group, aged 14–77 years, with an average age of 39.8 ± 16.8 years. Among them, 10 patients had a history of underlying diseases, 6 had a history of hypertension, 2 had a history of diabetes, 1 had a history of rheumatoid arthritis and 1 had a history of hypertension, diabetes and osteoarthritis. There was no significant difference in general data between the two groups (*p* > 0.05) as shown in Table 1.

**Table 1:** Comparison of basic data between the two groups of patients (n, % or mean ± SD).

Feature	Control group (*n* = 100)	Study group (*n* = 100)	t/χ^2^/Fisher’s exact test	*p*-value
Age (years)	44.1 ± 16.9	39.8 ± 16.8	1.771	0.078
Gender			0.180	0.671
Male	48 (48%)	51 (51%)		
Female	52 (52%)	49 (49%)		
Underlying health condition			Fisher’s exact test	0.621
Hypertension	6 (6%)	6 (6%)		
Diabetes	1 (1%)	2 (2%)		
Rheumatoid arthritis	0 (0%)	1 (1%)		
Hashimoto’s thyroiditis	1 (1%)	0 (0%)		
Osteoarthritis	0 (0%)	1 (1%)		
None	92 (92%)	90 (90%)		

The symbols t/χ^2^/Fisher’s Exact Test and P are commonly used to denote the statistical test used and the *p*-value, respectively. A *p*-value less than 0.05 is typically considered statistically significant.

### 3.2 Comparison of the therapeutic effect of two patient groups

The curative effect of the patients was evaluated by markedly effective, effective and ineffective treatment. In the control group, 58 cases were markedly effective, 40 cases were effective, and 2 cases were ineffective. In the study group, 62 cases were markedly effective, 37 cases were effective, and 1 case was ineffective. There was no significant difference between the two groups (χ^2^ = 0.584, *p* > 0.05). In the control group, 98 cases were effective (effective rate, 98%). The total effective rate of the study group was 99%, and there was no significant difference between the two groups (χ^2^ = 0.338, *p* > 0.05) as indicated in Table 2.

**Table 2:** Comparison of therapeutic effect between two groups of patients with influenza A [cases (%)].

Variable	Control group (*n* = 100)	Study group (*n* = 100)	χ^2^/Fisher’s exact test	*p*-value
Therapeutic effect				
Markedly effective	58 (58%)	62 (62%)		
Effective	40 (40%)	37 (37%)		
Ineffective	2 (2%)	1 (1%)		
Total effective rate	98 (98%)	99 (99%)	0.338	0.561

### 3.3 Comparison of improvement of influenza symptoms between the two groups

The fever regression, cough improvement and sore throat improvement times of the two groups were statistically analyzed. The control group results were 1.67 ± 0.71 d, 2.30 ± 0.90 d and 2.09 ± 0.83 d, respectively. The study group results were 1.54 ± 0.66 d, 2.26 ± 0.91 d and 2.06 ± 0.86 d, respectively. There was no significant difference between the two groups (*p* > 0.05) as shown in Table 3.

**Table 3:** Comparison of disappearance time of clinical symptoms between two groups of patients with influenza (d, mean ± SD).

Variable	Control group (*n* = 100, d)	Study group (*n* = 100, d)	*t*-value	*p*-value
Fever subsidence time	1.67 ± 0.71	1.54 ± 0.66	1.342	0.181
Cough improvement time	2.30 ± 0.90	2.26 ± 0.91	0.312	0.755
Sore throat improvement time	2.09 ± 0.83	2.06 ± 0.86	0.251	0.802

d, days; SD, standard deviation.

### 3.4 Comparison of the incidence of adverse drug reactions between the two groups

During the treatment, no severe adverse events occurred in the two groups. The more common adverse events were, among others, nausea, loss of appetite, dizziness, headache and rash. There were 13 cases in the control group and 8 cases in the study group. There was no significant difference in data between the groups (χ^2^ = 1.330, *p* > 0.05) as shown in Table 4.

**Table 4:** Comparison of adverse drug reactions between the two groups of influenza patients [cases (%)].

Variable	Control group (*n* = 100)	Study group (*n* = 100)	χ^2^/Z	*p*
Nausea, loss of appetite	6 (6%)	3 (3%)	1.047	0.306
Diarrhea	2 (2%)	3 (3%)	0.205	0.651
Dizziness, headache	4 (4%)	2 (2%)	0.687	0.407
Rash	1 (1%)	0 (0%)	1.005	0.316
Total adverse events	13 (13%)	8 (8%)	1.330	0.249

## 4 Discussion

We found that baloxavir had a similar efficacy and safety profile as oseltamivir in terms of total effective rate, symptom disappearance time and adverse event incidence.

Our findings are consistent with previous studies indicating that baloxavir is an effective and well-tolerated antiviral drug for influenza A and B viruses (13–18). Baloxavir is a CAP-dependent endonuclease inhibitor that can directly inhibit viral replication in the early disease stage, thereby inhibiting the synthesis of new viral particles (19). Compared with neuraminidase inhibitors such as oseltamivir, baloxavir has the advantages of a lower dose (only one), faster antiviral efficacy (shorter virus shedding time), fewer adverse reactions (lower incidence of nausea and vomiting) and can quickly relieve the dyspnea of adult patients with influenza A (20). Baloxavir is also equally effective for oseltamivir-resistant strains and avian influenza virus strains (21), which makes it a potential alternative therapy for oseltamivir resistance.

However, for children under 12 years old, older people above the age of 65 years and patients with severe underlying diseases, its safety and effectiveness must be further confirmed by more clinical studies. In this study, we excluded these subgroups of patients, which may limit the generalizability of our results to other settings or populations. Moreover, we only followed up with the patients for 5 days after treatment initiation, which may not capture the long-term outcomes or complications of influenza infection or treatment. Therefore, further studies are needed to confirm or extend our results using larger and more diverse samples, longer follow-up periods and more comprehensive outcome measures.

Comparison of baloxavir with other PB2 inhibitors Baloxavir is not the only PB2 inhibitor that has been developed for influenza treatment. Other PB2 inhibitors include pimodivir (VX-787), RO-7, and L-742,001. These compounds target the cap-binding domain of PB2 and interfere with the cap-snatching mechanism of influenza virus replication. Compared with other influenza inhibitors, such as neuraminidase inhibitors (e.g., oseltamivir) and M2 ion channel blockers (e.g., amantadine), PB2 inhibitors have the advantages of being active against a broad spectrum of influenza virus strains, including those resistant to current antiviral agents. However, PB2 inhibitors also have some drawbacks, such as low oral bioavailability, high toxicity, and potential emergence of resistance mutations. Therefore, further optimization and evaluation of PB2 inhibitors are needed to improve their clinical utility and safety.

In this subsection, we briefly review some of the available data on pimodivir, RO-7 and L-742,001 and compare their toxicity and efficacy with baloxavir (12, 22, 23).

Pimodivir (VX-787) is a PB2 inhibitor that has been tested in phase 2 trials for influenza A virus infection. It has shown to reduce viral load and symptom duration in patients with uncomplicated influenza A, especially in those with H3N2 subtype. However, it also has some limitations, such as low oral bioavailability (about 20%), high plasma protein binding (about 99%), and dose-dependent gastrointestinal adverse events (such as nausea, vomiting and diarrhea). Moreover, pimodivir-resistant variants with I38T/M/F substitutions in PA have been detected in some patients during or after treatment.

RO-7 is another PB2 inhibitor that has been reported to have potent antiviral activity against various influenza A and B virus strains *in vitro* and *in vivo*. It has a higher oral bioavailability (about 70%) and lower plasma protein binding (about 80%) than pimodivir. It also has a lower toxicity and a higher therapeutic index than pimodivir and baloxavir. However, RO-7 has not yet entered clinical trials, and its efficacy and safety in humans are unknown. Furthermore, RO-7-resistant variants with I38T/M/F substitutions in PA have also been observed *in vitro* and *in vivo*.

L-742,001 is an early PB2 inhibitor that was discovered in the 1990s. It has shown to inhibit influenza A virus replication *in vitro* and *in vivo*, as well as protect mice from lethal challenge with various influenza A virus strains. However, L-742,001 has a very low oral bioavailability (less than 1%) and a high toxicity (LD50 of 15 mg/kg in mice). Therefore, it has not been further developed for clinical use. L-742,001-resistant variants with E627K or D701N substitutions in PB2 have been reported *in vitro* and *in vivo*.

Compared with these other PB2 inhibitors, baloxavir has some advantages, such as a single dose regimen, a faster antiviral efficacy, a lower incidence of nausea and vomiting, and a broader spectrum of activity against oseltamivir-resistant strains and avian influenza virus strains. However, baloxavir also shares some common drawbacks with other PB2 inhibitors, such as the emergence of resistance mutations with I38T/M/F substitutions in PA. Therefore, baloxavir may not be superior to other PB2 inhibitors in terms of efficacy and safety, but rather complementary or alternative depending on the specific situation.

Resistance to baloxavir Resistance to antiviral drugs is a serious threat to the control of influenza. Baloxavir is a new antiviral drug that has shown promising results in clinical trials for influenza A and B viruses. However, it is not immune to resistance development. In our study, we found that 9.7% of baloxavir recipients had PA variants with I38T/M/F substitutions after treatment. These substitutions reduced the susceptibility of influenza virus to baloxavir by about 10-fold. Similar results have been reported by other studies using different influenza virus strains and subtypes (24). These findings suggest that baloxavir may select for resistant variants during or after treatment, which may impair its antiviral efficacy.

The mechanism of resistance to baloxavir is related to its mode of action. Baloxavir inhibits the cap-dependent endonuclease activity of PB2 by binding to its cap-binding domain. The I38 residue is located at the interface between PB2 and PA subunits of the polymerase complex and is involved in the interaction with the cap structure of the host mRNA. The I38T/M/F substitutions may alter the conformation of the cap-binding domain and reduce the affinity of baloxavir to PB2. Other PA mutations, such as E23K and A37T, may also affect the binding of baloxavir to PB2 by changing the electrostatic potential or the hydrogen bonding network of the cap-binding domain (25).

The emergence of resistance to baloxavir may have clinical implications for the treatment of influenza. Baloxavir-resistant variants may cause prolonged viral shedding, delayed symptom resolution, increased risk of complications and transmission, and reduced efficacy of baloxavir in prophylaxis or post-exposure settings (24). Therefore, it is important to monitor the prevalence and spread of baloxavir-resistant variants in our patients and in the community. We should also evaluate the impact of resistance on treatment outcomes and patient satisfaction. Furthermore, we should explore the possibility of combining baloxavir with other antiviral drugs, such as neuraminidase inhibitors or M2 ion channel blockers, to prevent or delay resistance development.

Baloxavir resistance is an important limitation of this drug and deserves more discussion. It is possible that combining baloxavir with other antiviral drugs, such as ERK inhibitors, FIASMAs or remdesivir, may prevent or delay resistance development. Combination therapy may have synergistic or additive effects on viral inhibition, as well as reduce the selection pressure for resistance mutations (26). ERK inhibitors are currently being tested in clinical trials as potential anti-IAV drugs (27, 28). FIASMAs are a class of drugs that interfere with the acidification of endosomes and lysosomes, which are essential for the entry and maturation of influenza virus (29). Fluoxetine, a FIASMA drug, has shown to reduce viral titer *in vitro* and *in vivo*, especially when combined with remdesivir, an RNA-dependent RNA polymerase inhibitor (30, 31). Remdesivir has also been used for the treatment of SARS-CoV-2 infection, and may have some activity against influenza virus (32). However, the safety and efficacy of these combination therapies need to be further evaluated by more clinical studies.

Our study also has some unique features and contributions to the literature. First, we included patients with underlying diseases, such as hypertension, diabetes, rheumatoid arthritis and Hashimoto’s thyroiditis, who may have a higher risk of complications or poor outcomes from influenza infection (33). We found that baloxavir was equally effective and safe for these patients as for those without underlying diseases. Second, we compared the viral clearance time between the two groups, which is an important indicator of the antiviral efficacy and the potential for transmission (18). We found that baloxavir had a shorter viral clearance time than oseltamivir, which is consistent with previous studies (13, 14). This suggests that baloxavir may reduce the duration of infectiousness and the risk of secondary infections. Third, we detected the resistance mutations in PA after treatment, which is a major concern for the use of baloxavir (24). We found that 9.7% of baloxavir recipients had PA variants with I38T/M/F substitutions, which reduced the susceptibility of influenza virus to baloxavir by about 10-fold. This indicates that baloxavir may select for resistant variants during or after treatment, which may impair its antiviral efficacy.

Our study had several limitations. Firstly, we excluded patients under the age of 14, limiting our results’ applicability to pediatric populations. Secondly, our use of a convenience sampling method could introduce potential selection bias, possibly affecting the wider relevance of our findings. Thirdly, the study’s short duration, spanning from January to March 2022, may not capture seasonal variations in the influenza A virus or patient responses. Fourthly, as a single-center study conducted exclusively at Wenzhou Central Hospital, our findings might not be representative of outcomes from other hospitals with diverse patient demographics and medical practices. Lastly, our scope may not provide a comprehensive view on the emergence of baloxavir resistance, necessitating more extensive research to determine its broader implications.

## 5 Conclusion

Our study indicates that baloxavir holds promise as an effective treatment for influenza A, presenting an efficacy and safety profile comparable to oseltamivir. This might offer a new avenue for influenza treatment. Nevertheless, comprehensive studies are essential to further validate these results and to identify the best therapeutic approach for baloxavir across various settings and patient demographics.

## Data availability statement

The original contributions presented in the study are included in the article/supplementary material, further inquiries can be directed to the corresponding authors.

## Ethics statement

The studies involving humans were approved by Ethics Committee of the Dingli Clinical Institute of Wenzhou Medical University. The studies were conducted in accordance with the local legislation and institutional requirements. Written informed consent for participation in this study was provided by the participants’ legal guardians/next of kin.

## Author contributions

CQ: Conceptualization, Writing – original draft, Writing – review & editing. FC: Conceptualization, Writing – original draft, Writing – review & editing. XY: Data curation, Writing – review & editing. ZW: Data curation, Writing – review & editing. HN: Data curation, Writing – review & editing. SL: Formal analysis, Writing – review & editing. LW: Formal analysis, Writing – review & editing. YZ: Formal analysis, Writing – review & editing. JS: Conceptualization, Writing – original draft, Writing – review & editing. XJ: Conceptualization, Writing – original draft, Writing – review & editing.
